# Graft Engineering and Adoptive Immunotherapy: New Approaches to Promote Immune Tolerance After Hematopoietic Stem Cell Transplantation

**DOI:** 10.3389/fimmu.2019.01342

**Published:** 2019-07-10

**Authors:** Alice Bertaina, Maria Grazia Roncarolo

**Affiliations:** ^1^Division of Stem Cell Transplantation and Regenerative Medicine, Department of Pediatrics, Stanford School of Medicine, Stanford, CA, United States; ^2^Institute for Stem Cell Biology and Regenerative Medicine, Stanford School of Medicine, Stanford, CA, United States

**Keywords:** immune tolerance, gamma delta (γδ) T cells, Treg - regulatory T cells, CAR (chimeric antigen receptor) T cells, haploidentical allogeneic hematopoietic stem cell transplantation

## Abstract

Allogeneic hematopoietic stem cell transplantation (HSCT) is a curative therapeutic option for a wide range of immune and hematologic malignant and non-malignant disorders. Once transplanted, allogeneic cells have to support myeloid repopulation and immunological reconstitution, but also need to become tolerant to the host via central or peripheral mechanisms to achieve the desired therapeutic effect. Peripheral tolerance after allogeneic HSCT may be achieved by several mechanisms, though blocking alloreactivity to the host human leukocyte antigens while preserving immune responses to pathogens and tumor antigens remains a challenge. Recently uncovered evidence on the mechanisms of post-HSCT immune reconstitution and tolerance in transplanted patients has allowed for the development of novel cell-based therapeutic approaches. These therapies are aimed at inducing long-term peripheral tolerance and reducing the risk of graft-vs-host disease (GvHD), while sparing the graft-vs-leukemia (GvL) effect. Thus, ensuring effective long term remission in hematologic malignancies. Today, haploidentical stem cell transplants have become a widely used treatment for patients with hematological malignancies. A myriad of *ex vivo* and *in vivo* T-cell depletion strategies have been adopted, with the goal of preventing GvHD while preserving GvL in the context of immunogenetic disparity. αβ T-cell/CD19 B-cell depletion techniques, in particular, has gained significant momentum, because of the high rate of leukemia-free survival and the low risk of severe GvHD. Despite progress, better treatments are still needed in a portion of patients to further reduce the incidence of relapse and achieve long-term tolerance. Current post-HSCT cell therapy approaches designed to induce tolerance and minimizing GvHD occurrence include the use of (i) γδ T cells, (ii) regulatory Type 1 T (Tr1) cells, and (iii) engineered FOXP3^+^ regulatory T cells. Future protocols may include post-HSCT infusion of allogeneic effector or regulatory T cells engineered with a chimeric antigen receptor (CAR). In the present review, we describe the most recent advances in graft engineering and post-HSCT adoptive immunotherapy.

## Introduction

Successful allogeneic hematopoietic stem cell transplantation (HSCT) requires the development of immune tolerance toward both the donor and host allogeneic antigens. Induction of immune tolerance can prevent T-cell mediated graft-rejection and graft-vs-host disease (GvHD), which cause severe pathology in HSCT recipients. Current approaches to prevent rejection and GvHD after HSCT primarily rely on pharmacological immune suppression, either prior to or after HSCT. These approaches are limited by acute and long-term drug toxicity, lack of antigen specificity, and the requirement for long-term therapy, which often leads to severe complications. Recent progress in understanding the mechanism of action of alloreactive and regulatory cell populations has led to the use of specific cell subsets to prevent/treat graft rejection and GvHD and induce immune tolerance. In hematologic malignancies maintaining effective anti-tumor control while inducing sustained immune tolerance is critical to survival following allogeneic HSCT.

In the past decade, several new graft-engineering approaches have been explored to reduce the risk of life-threatening GvHD, while retaining the effector cells that mediate infection control and graft-vs-leukemia (GvL). Concurrently, post-HSCT adoptive cell therapies have been used with increasing frequency to induce tolerance and reduce the risk of leukemia recurrence. This review will summarize the results of these new approaches in patients with hematological malignancies.

## Recent Advances in Graft Manipulation: The Role of HLA-Haploidentical HSCT

Allogeneic HSCT from an HLA-matched donor, either related or unrelated, has been widely employed to treat patients with both malignant and non-malignant disorders (1). Only 25% of patients who are candidates to receive allogeneic HSCT have an HLA-identical sibling. Suitable, unrelated donors (UD) can be identified for <60% of the remaining patients in need (2). The likelihood of finding an appropriate UD varies among racial and ethnic groups, with the probability of identifying an appropriate donor being highest among whites of European descent (75%) and lowest among blacks or those of South or Central American descent (16%) (3). As such, a related full-haplotype mismatched donor (haploidentical) as an alternative source of hematopoietic stem cell (HSC), is highly attractive, as virtually all patients have a readily available haploidentical family member who can serve as an HSC donor (2, 4, 5). Despite many advantages associated with haploidentical (haplo-) HSCT, in the past the widespread use of this procedure was hampered by severe clinical complications due to bidirectional alloreactivity toward incompatible HLA molecules, including high rates of graft-rejection and severe GvHD. Since donor-derived T lymphocytes contained in the graft are the major mediators of severe GvHD in haplo-HSCT, several approaches have been explored to deplete T cells from the graft prior to or post-infusion. Over the past 10 years, the clinical use of haploidentical donors has gained traction thanks to the use of T-cell depleted peripheral blood stem cells (PBSC) or of unmanipulated (either bone marrow -BM- or PBSC) grafts followed by high-dose post-transplant cyclophosphamide (PTCY) (6–9). The very first *ex vivo* T-cell depleted haplo-HSCTs using soybean agglutinin and rosette formation sheep red blood cells were performed in children with primary immunodeficiencies (10). As of today, hundreds of Severe Combined Immune Deficiency (SCID) patients have been transplanted worldwide using an HLA-haploidentical related donor, with a high rate of long-term, partial or complete, immune reconstitution (11). Initially, these encouraging outcomes were not replicable in leukemia patients, in whom haplo-HSCT was associated with an unacceptably high incidence of graft failure (12). Since then, several preclinical studies have led to a variety of promising techniques to diminish the intense alloreactivity in haplo-HSCT for hematological malignancies. These new approaches have yielded high rates of successful engraftment, effective GvHD control and favorable outcomes. Retrospective analyses of adult cohorts reported in the last decade have demonstrated similar survival after haplo-HSCT, HLA-matched-related, or HLA-matched-unrelated HSCT in leukemia patients (13, 14).

The unmanipulated haploidentical approach, pioneered by the group of Fuchs EJ and Luznik L, relies on the use of PTCY. This drug targets the early proliferation of both donor and recipient alloreactive T cells that occurs in the first few days after HSCT (15). Indeed, cyclophosphamide mediates *in vivo* depletion of both donor and recipient alloreactive cells while sparing quiescent non-alloreactive T cells, when given in the 72 h window after T-cell replete HSCT (either BM or PBSC). This method promotes engraftment and reduces the risk of severe acute GvHD. Pilot studies in adults conditioned with a non-myeloablative (NMA) preparative regimen and transplanted with BM cells showed 90% engraftment with very low incidence of both acute and chronic GvHD (16). Subsequent studies in haplo-HSCT using myeloablative conditioning and PTCY reported better control of leukemia with no significant increase in GvHD or Non-relapse mortality (NRM) (17, 18). The use of PBSC as graft source instead of BM led to some increase in acute GvHD incidence, but similar outcomes in terms of engraftment and NRM (19). Overall, these studies have established PTCY-based haplo-HSCT as a frontrunner for alternate donor HSCT in adults, prompting selection of PTCY-based haplo-HSCT over matched UD (MUD) or umbilical cord blood (UCB) HSCT (14) for many patient.

While this strategy has been extensively investigated in adult patients, results on the use of unmanipulated haplo-HSCT in the pediatric population have only recently been published (20–22). Early results of GvHD prevention are encouraging, though limited information on follow-up results is available.

### *Ex-vivo* T-Cell Depletion in Haploidentical HSCT: The Evolution

Pioneering studies in adults demonstrated that infusion of “*megadoses*” of purified CD34^+^ cells can prevent both graft rejection and severe GvHD in adult haplo-HSCT recipients (12, 23). In a pilot study of adults with acute leukemia, Aversa et al. used a combination of donor BM and G-CSF-mobilized PBSC. This allowed for the collection of 7–10 times more hematopoietic progenitor cells compared to BM allografts alone. Allografts were T-cell depleted using soybean agglutination and erythrocyte rosetting. No post-transplant immunosuppression was given. Engraftment rate was above 90%, with a cumulative incidence of both grade II–IV acute and chronic GvHD below 10%. In this study, only two patients relapsed, but NRM occurred in 9 out of 17 patients (23). The method was then further improved by immunomagnetic selection of CD34^+^ cells, which drastically reduces the T- and B-cell content in the graft, allowing the infusion of more than 10 × 10^6^/kg CD34^+^ cells, with a mean CD3^+^ cell infusion of a 3 × 103/kg. In this seminal study, Aversa et al. showed sustained engraftment in 41/43 adult and pediatric patients (age range 4–53 years) with advanced leukemia, without acute or chronic GvHD and a long term disease-free survival (DFS) rate of 28% (12). Although the administration of HSC “*megadoses*” addressed the rejection problem, removal of T cells from the graft entailed prolonged lymphopenia and delayed immune reconstitution for patients, with low CD4^+^ T cells persisting for more than a year after transplantation. As a result, the NRM was 40%, with two thirds of these deaths due to opportunistic infections. After these initial studies, Martelli et al. reported that freshly isolated donor-derived regulatory T cells (Tregs), coinfused with conventional T cells (Tcons), protect recipients against GvHD (24). In 2014, the same group investigated whether Treg-Tcons adoptive immunotherapy prevents post-HSCT leukemia relapse (24). Forty-three adults with high-risk acute leukemia conditioned with a total body irradiation (TBI)-based regimen received CD34^+^ cells, Tregs and Tcons without post-HSCT immunosuppression. Ninety-five percent of patients achieved full-donor engraftment but 15% of them developed grade II-IV acute GvHD. The rate of DFS was 56% at a median follow-up of 46 months. Cumulative incidence (CI) of relapse (REL) (5%) was significantly better than in historical controls. These results demonstrate the immunosuppressive potential of Tregs in preventing GvHD without loss of GvL activity. Humanized murine models have since provided insights into the mechanisms underlying the separation of GvL from GvHD, with the effect of GvL being largely due to unopposed Tcons alloantigen recognition in BM.

Aversa et al. have also performed an extensive analysis of using CD34^+^ positively selected haplo-HSCT combined with rigorous T-cell depletion of the graft and the use of PTCY (25) in both a pre-clinical model and three patients. In this report, the authors demonstrated that, in mice treated with a NMA conditioning regimen, coupling the power of high-dose PTCY with the infusion of T-cell depleted HSC “*megadoses*” can be a suitable option for overcoming graft rejection. This approach was then evaluated in two patients with multiple myeloma and one patient with Hodgkin lymphoma. At a 25 month follow up, the first myeloma patient exhibited full donor chimerism in the myeloid- and B-cell lineages and mixed chimerism in the T-cell lineage. Conversely, the second myeloma patient failed to attain chimerism. Notably, the low toxicity of this protocol enabled a subsequent successful fully myeloablative haplo-HSCT in this patient. The third patient was conditioned with slightly higher TBI, resulting in prompt engraftment. All patients currently remain in remission without GvHD (25). These preliminary data lay the foundation for a novel and safer NMA haplo-HSCT, a potential platform for immune tolerance induction for either cell therapy and/or solid organ transplantation.

Recently, another approach to selective T-cell depletion has been developed, based on removal of CD45RA^+^ *naïve* T lymphocytes, while retaining CD45RO^+^ memory T cells. The rationale for this strategy is based on experimental data demonstrating that mouse CD4^+^ memory T cells, as well as effector memory CD8^+^ T cells, are devoid of GvHD reactivity (26). A recent study of 17 patients with high risk hematologic malignancies detailed the results of performing CD45RA^+^ depleted haplo-HSCT following a novel TBI- and serotherapy-free reduced-intensity conditioning (RIC) regimen. Remarkable depletion of CD45RA^+^ T cells and B cells, with preservation of abundant memory T cells, was achieved in all 17 grafts. No infection-related mortality has been reported. Despite the infusion of a median of >100 × 10^6^ haploidentical T cells, no patient experienced acute GvHD. However, 6/17 developed symptoms of chronic GvHD (27). This finding may be explained by the fact that the CD45RA^+^-depleted fraction contained both T effector-memory (EM) cells and T central-memory (CM) cells which may mediate chronic GvHD (28).

### αβ T-Cell/CD19 B-Cell Depleted Haploidentical Transplant: A New Source of Effector and Tolerogenic Cells?

Despite encouraging clinical data using “*megadoses*” of purified CD34^+^ cells, extensive lymphoid cell depletion results in an increased risk of opportunistic infections, especially in the first months after HSCT. To reduce the risk of infection and leukemia recurrence, a new strategy of graft manipulation has been implemented based on the selective elimination of αβ T cells and CD19 B cells (αβhaplo-HSCT) (29). This refined technique of graft engineering reduces complications associated with delayed immune recovery observed in the purified CD34^+^ haplo-HSCT approach. With the αβhaplo-HSCT cell selection approach, it is possible to transfer HSCs and committed hematopoietic progenitors to the recipient donor, as well as mature natural killer (NK) and γδ T cells (30, 31). We first reported promising clinical results using αβhaplo-HSCT in children with life-threatening non-malignant disorders (32). More recently, single-center experiences in pediatric patients with malignancies have been published (33–35). These studies show that the risk of NRM and leukemia relapse are comparable to those in HLA-identical siblings or UD-HSCT. Moreover, patients receiving αβhaplo-HSCT have a lower risk of acute and chronic GvHD, leading to better GvHD-free/relapse-free survival (GRFS). These data were confirmed in a multicenter retrospective analysis which compared 98 αβhaplo-HSCT recipients with 245 UD-HSCT (36). This study definitively established that αβhaplo-HSCT is an equally effective option to UD-HSCT for children with acute leukemia lacking a sibling donor.

The NK and γδ T cells which are still present in the αβhaplo-HSCT might facilitate engraftment and reduce the risk of both infections and leukemia recurrence (30, 31). While T cells carrying the αβ TCR are responsible for GvHD (37, 38), γδ T cells have no alloreactive capacity (39), but do have important anti-infectious (40) and anti-leukemia effects (41–44). Thanks to this novel graft manipulation approach, patients can immediately benefit from donor NK cells contained in the graft that are able to fully exert their activity in the 6–8 weeks after transplant, before mature KIR^+^ NK cells differentiate from CD34^+^ cells (45, 46). The infusion with the graft of these different lymphoid cell subsets explains that NRM and LFS are superimposable in αβhaplo-HSCT and allelic matched UD (MUD)-HSCT (36). The large number of effector cells infused with the graft, along with a “*megadose*” of HSCs combined with the fully myeloablative conditioning regimen, might explain the remarkably low incidence of graft failure (2%) observed in αβhaplo-HSCT. The absence of pharmacological post-HSCT GvHD prophylaxis and the use of a fully myeloablative conditioning regimen could also be responsible for the lower incidence of relapse (CI of REL 29%, 95% CI 20–42) when compared with other previously published studies (33, 34, 36).

A randomized, prospective trial between haplo-HSCT (either αβhaplo-HSCT or PTCY) and MUD recipients is expected to start in the next 18 months. Thanks to these recent results, αβhaplo-HSCT is becoming the first choice for T-cell depletion in HSCT for pediatric patients affected by hematological malignancies.

## Immunoregulatory Cells to Achieve Transplantation Tolerance and Graft-vs-Leukemia Effect

### Anti-infectious, Anti-leukemic, and Tolerogenic Properties of γδ T Cells

γδ T cells are a small subset of T lymphocytes in the peripheral blood, but constitute a major T-cell population in tissues (47). These T cells mediate both adaptive and rapid, innate-like immune response, playing multiple roles in the initiation and effector phases of immune reactions. In contrast to conventional αβ T cells, the available number of germline genes coding for T-cell receptor (TCR) variable elements of γδ T cells is very small. There is a preferential localization of γδ T cells expressing given Vγ and Vδ genes in certain tissues. γδ T cells play an important role in the successful clinical outcome of αβhaplo-HSCT in pediatric patients with high-risk leukemias because they can recognize tumor cells without the need for major histocompatibility complex (MHC) presentation (47) and have potent anti-leukemia activity in the absence of relevant GvHD-inducing effect (35, 36, 48). The results of recent studies suggest advantageous effects of elevated γδ T cell immune recovery after HSCT in terms of infections, GvHD and overall survival (43, 49). Despite this, further clarification is needed to properly assess γδ T-cell potential in HSCT. This includes investigation of tissue biopsies (i.e., gut, liver, skin) and peripheral blood samples from patients with GvHD to better determine and differentiate the effects of various γδ T-cell subtypes.

The main subset of circulating γδ T cells express the Vδ2 chain associated with Vγ9 (i.e., Vγ9Vδ2). γδ T cells bearing the Vδ1 chain are a minor subset. Both subsets share antitumor properties, but Vδ1 cells also reside within epithelial tissues and may undergo selective expansion in transplanted patients upon cytomegalovirus (CMV) reactivation. Human Vγ9Vδ2 T cells recognize phosphorylated metabolites (phosphoantigens) that are secreted by many pathogens, but are also overexpressed by tumor cells, explaining why these cells play a role in both anti-infectious and anti-tumor immune responses. Similarly, the recently reported ability of human non-Vδ2 γδ T cells to recognize endothelial protein C receptor provides a link between immunity against epithelial tumor cells and CMV-infected endothelial cells (50).

To better understand the role of γδ T cells in αβhaplo-HSCT, we recently investigated their reconstitution kinetics in 27 children, 15 of whom had leukemia (51). Immunophenotypic characterization of peripheral blood mononuclear cells at one, three, and six months after HSCT revealed an initial abundance of γδ T cells, followed by a progressive predominance of αβ T cells. As in healthy donors, γδ T cells included three different populations: Vδ2, Vδ1 and, to a lesser extent, Vδ2^−^/Vδ1^−^ T cells. The relative proportions of the different Vδ2 and Vδ1 populations remained stable over time and were similar to those detected in the donor's HSCs. Naïve Vδ2 cells increased significantly between 20 days and three months after αβhaplo-HSCT, suggesting that circulating γδ T cells in transplanted patients consisted of not only mature cells derived from the graft, but also of cells differentiating from donor's HSCs (51). In patients given αβhaplo-HSCT, CMV specific cells were predominant in the Vδ1 T-cell subset, in contrast to healthy donors (51). Patients experiencing CMV reactivation displayed a significant expansion of the Vδ1 T-cell subset with a cytotoxic EM phenotype, which was absent in patients without CMV reactivation. These CMV-driven Vδ1 T cells killed *in vitro* primary acute lymphoblastic (ALL) and acute myeloid (AML) blasts more efficiently than Vδ1 T cells from patients that did not reactivate CMV infection, suggesting that CMV infection promotes both expansion and activation of Vδ1 T cells (51). These findings are consistent with those in kidney transplant, demonstrating expansion of Vδ2^−^ γδ T cells displaying an EM phenotype and exerting cytotoxic function upon CMV infection (52). Interestingly, the expansion of Vδ1 and Vδ1^−^/Vδ2^−^ T cells with a restricted TCR repertoire was observed during CMV infection, which is indicative of antigen-driven selection (53).

We also showed that Vδ2 T cells from patients who received αβhaplo-HSCT expanded *in vitro* upon incubation with zoledronic acid (ZOL), which promoted the acquisition of an EM phenotype and potentiated the cytotoxic activity against primary leukemic blasts. This activity is dependent on the levels of phosphoantigens expressed by leukemia cells and on TCR Vγ9 mediated recognition (51). Indeed, lytic capacity of γδ T cells is strongly enhanced by sensitizing the leukemic target cells with ZOL. Based on these *in vitro* results, a clinical study investigating the effect of ZOL infusion was performed in 43 pediatric recipients of αβhaplo-HSCT (44). Here, ZOL was infused every 28 days. The treatment was safe and well-tolerated, and, when administered three or more times, improved overall survival. The first treatment with ZOL induced the differentiation of Vδ2 T cells, switching them from a CM to an EM phenotype. Such maturation corresponded with increased Vδ2 T-cell mediated cytotoxicity against primary leukemia cells irrespective of their phosphoantigen expression. Proteomic analyses identified an anti-proliferative effect of infused ZOL on total γδ T cells consistent with the decrease in Vδ2 T cells, starting three months after HSCT. Such an effect was already evident after the first ZOL infusion and was further boosted by subsequent infusions. The percentage of Vδ1 T cells increased during ZOL infusions regardless of CMV reactivation (44). Altogether, these results suggest that maintenance and activation of γδ T cells after αβhaplo-HSCT improve long-term LFS. This approach may also potentiate the anti-leukemic activity of endogenous Vδ2Vγ9 T cells. Future prospective controlled clinical trials will determine if this approach leads to significant clinical benefit. In addition, future studies aimed at deeply understanding the fine mechanisms whereby γδ T cells, especially theVδ1 subset, recognize malignant and virus-infected cells will assist in uncovering the therapeutic potential of γδ T cells for haplo-HSCT.

Recent studies further suggest that some subsets of γδ T cells can have regulatory activity and antigen-presenting capacity, though their functional plasticity and the extent of γδ T cell ligand diversity have not yet been determined. Although they are FOXP3 negative, γδ T cells strongly suppress T helper cell proliferation in an IL-2-independent mechanism and produce high amounts of TGF-β. We are currently exploring whether γδ T cells can regulate the immune response mediated by αβ T cells after HSCT, especially in the context of immunogenetic disparity, such as in haplo-HSCT.

The suppressive function of human γδ T cells was first described in 1989 by Patel and colleagues (54). Since then, the regulatory role for γδ T cells have been reported in several pre-clinical and clinical studies (55–58). Both Vδ1 and Vδ2 T-cell subsets may exhibit regulatory properties, albeit in different settings (58).

Drobyski et al. showed that the co-administration of γδ T cells and naive αβ T cells at the time of MHC-mismatched HSCT infusion exacerbates GvHD, in comparison to the administration of naive αβ T cells alone. Conversely, when the infusion of naive αβ T cells was delayed for 2 weeks after HSCT, the survival of mice transplanted with BM and with activated γδ T cells was increased compared to that of mice given BM cells alone. These results indicate that only activated γδ T cells have a modulatory ability on effector αβ T cells to prevent GvHD after HSCT (59). The mechanism of suppression used by γδ T cells remains somewhat uncertain. Kuhl et al. claim that the regulatory activity is mediated by the immunosuppressive cytokines TGF-β1 and IL-10, which are secreted by γδ T cells after anti-CD3/CD28 mAb stimulation. They showed that activated γδ T cells secreted significantly more TGF-β1 than conventional CD4^+^CD25^+^ Tregs (60). They also quantified the relative TGF-β1 mRNA content in different γδ T-cell subsets isolated from peripheral blood. The Vδ1 cell subset showed increased TGF-β1 mRNA content compared with Vδ2 T cells in six out of seven donors, suggesting greater suppressive capacity of Vδ1 than Vδ2 and αβ T cells. Subsequently, Li and colleagues demonstrated that TGF-β1-stimulated CD25^+^CD27^+^Vδ1 γδ T cells exert a suppressive effect on naïve CD4^+^ T cells similar to classical CD4^+^FOXP3^+^ Tregs and that this mechanism is cell-cell contact dependent. Peters et al. demonstrated that co-culturing of Vδ cells with responder cells (CD25-depleted CD4^+^ αβ T cells) leads to upregulation of CD80, CD86, and PDL-1 on stimulated Vδ2 γδ T cells, which then interact with CTLA-4 or PD-1 on responder cells, leading to their suppression (56). In this study, the immunosuppressive capacity of Vδ2 γδ T cells was abrogated by Toll-like-receptor (TLR) 2 ligands, which correlated with increased phosphorylation of Akt and NFκB in αβ T cells and down-regulation of inhibitory molecules such as PD-1 and CTLA-4. Based on these studies, it can be hypothesized that the elimination of suppressive γδ T cells from cells used for adoptive immunotherapy in cancer patients could be useful in boosting antitumor effects.

Overall, γδ T cells play an important role in the successful clinical outcome of HSCT, but may act as a double-edged sword with both effector and regulatory functions dependent on the subset of cells and the environment they are in. Manufacturing of a cell product containing γδ T-cell subsets with anti-infectious and anti-leukemia activity, but lacking regulatory function, could have future clinical applications in haplo-HSCT.

### Regulatory Type 1 T (Tr1) Cells: Clinical Application

Mechanisms underlying tolerance after allogenic HSCT consist of peripheral clonal deletion or active suppression mediated by regulatory cells such as T, B, NK, and mesenchymal stromal regulatory cells. Among Tregs, CD4^+^ and CD8^+^ or double negative T cells have been described extensively in literature (59). Tregs are an important component of the immune system involved in dampening immune reactions and inducing tolerance (61). Due to their efficacy as immune modulators in several pre-clinical models, the clinical applications of Tregs have been extensively explored. Several types of CD4^+^ Tregs have been identified, including the forkhead box P3 (FOXP3)-expressing Tregs (FOXP3^+^ Tregs) and regulatory type 1 T (Tr1) cells (62). For the purpose of this review, we will focus on Tr1 cells and their clinical applications in the context of allogeneic HSCT, as well as on engineered FOXP3^+^ Tregs.

Studies in patients with SCID and β-thalassemia who became chimeric post-HSCT demonstrated that, although clonal deletion of alloreactive T cells might occur, peripheral tolerance mediated by Tr1 cells is crucial for active suppression of effector T-cell alloreactivity (62–64). CD4^+^ Tr1 cells are defined and distinct from Th1, Th2, Th3, and Th17 cells, based on their unique pattern of cytokine production. They produce high levels of IL-10, TGF-β, low levels of IL-2, variable levels of IL-5 and IFN-γ in the absence of IL-4 and IL-17 (65). Tr1 cells have specific metabolic requirements that distinguishes them from FOXP3^+^ Tregs: Tr1 cells depend on glycolysis and are inhibited by hypoxia and extracellular ATP (66), while peripheral FOXP3^+^ Tregs depend on fatty acid oxidation (67). We have demonstrated that CD49b LAG-3 are good markers for a subset of memory Tr1 cells (68). The vast majority of memory CD4^+^CD49b^+^LAG-3^+^ T cells secrete large amounts of IL-10 but not significant levels of IL-4 and IL-17, do not constitutively express FOXP3, and display regulatory activity both *in vitro* and *in vivo*. Both CD49b and LAG-3 are stably expressed on functional human Tr1 cell clones. CD49b is expressed on Tr1 cells regardless of their activation state, whereas LAG-3 is expressed on Tr1 cells upon activation when the cells produce IL-10 and display suppressor activity. CD49b and LAG-3 have been used to identify Tr1 cells in the peripheral blood of healthy donors and tolerant patients. CD49b and LAG-3 can be used to track Tr1 cells in cell products generated *in vitro* (68). Tr1-cell mediated suppression is mainly driven by the secretion of IL-10 and TGF-β. Tr1 cells require activation via their TCR to mediate suppression, but once activated, they mediated bystander suppression against other antigens (62).

Tr1 cells were first identified and characterized in SCID patients who were immune reconstituted after HSCT from HLA mismatched donors (63, 64). These patients developed spontaneous split chimerism, with T and NK cells of donor origin and B and professional APC of host origin in the absence of GvHD. Single cell cloning of T cells from the peripheral blood of these chimeras leads to the isolation of donor derived CD4^+^ and CD8^+^ T-cell clones specific for the HLA antigens of the host (64). These data indicate that alloreactive T cells are not deleted from the T-cell repertoire of the patients. A significant number of these T-cell clones produce high levels of IL-10 when activated with the host alloantigens *in vitro* (63). These findings correlate with the absence of GvHD and a state of active tolerance between host and donor cells. Similar data was obtained from β-thalassemic patients who developed persistent mixed chimerism following HSCT (69). The presence of Tr1 cells *in vivo* was confirmed by detection of CD49b^+^ and LAG-3^+^ T cells at higher frequency in the peripheral blood mononuclear cells (PBMC) of these tolerant patients compared to normal donors' PBMC (68). Moreover, high amounts of IL-10 production were detected *ex vivo* by PBMCs of patients with persistent mixed chimerism. High levels of IL-10 production and presence of Tr1 cells were not detected in patients with complete donor chimerism, suggesting that chronic allo-antigen stimulation by mismatched host APCs plays a role in Tr1 cell induction *in vivo* (69). Overall, these results indicate that IL-10 and Tr1 cells are associated with long-term, spontaneously established, tolerance, suggesting Tr1-based cell therapy can be used to promote tolerance in HSCT (70). Several protocols have been developed to generate Tr1 cells *in vitro* that are suitable for *in vivo* use. Although IL-10 is indispensable for Tr1 cell induction, efficient *in vitro* production of Tr1 cells also requires APC. Tr1 cell induction *in vitro* is optimal when IL-10-producing human dendritic cells (DC-10) are used as APC (71). DC-10 are monocyte-derived dendritic cells generated *in vitro* in the presence of exogenous IL-10 in addition to GM-CSF and IL-4, which are required for *in vitro* differentiation of mature myeloid derived DC (71). DC-10 are CD14^+^, CD16^+^, CD11c^+^, CD11b^+^, HLA-DR^+^, CD83^+^, CD1a^−^, CD1c^−^, express the Ig-like transcripts (ILT)2, ILT3, ILT4, and HLA-G antigen and display high levels of CD40 and CD86 and up-regulate CD80 following differentiation *in vitro*. Stimulation of CD4^+^ cells with allogeneic DC-10 and IL-10 is efficient in generating a Tr1 -cell product that suppresses antigen-specific proliferative responses of autologous CD4^+^ T cells (68, 72).

A first clinical trial aimed at prevention of GvHD and establishment of immunological tolerance after haplo-HSCT with purified CD34^+^ cells in adult with high risk leukemias was carried out using donor Tr1 cells specific for host alloantigens (73). Adaptively infused donor T cells were primed by the host monocytes as APC and IL-10 for a short period of time. The method to “instruct” the Ag-specific T cells to differentiate into Tr1 cells and generate allo-specific IL-10-anergized T cells (IL-10 DLI) was validated in good-manufacturing-practice (GMP) (74, 75). Following donor myeloid engraftment in 12 patients post-CD34^+^ purified haplo-HSCT, these cells were infused at the dose of 10^5^ CD3^+^ T cells/kg. Patients experienced only mild to moderate GvHD and had a rapid normalization of their lymphocyte counts. Moreover, they showed a normal polyclonal TCR repertoire and presented a good *in vitro* T-cell response against viral antigens and mitogens. Donor T cells remained hyporesponsive to host alloantigens *in vitro*, and cells with the Tr1-cell specific phenotypic markers CD49b and LAG3 were observed to increase over time in the peripheral blood. Four patients benefited from this adoptive immunotherapy and fully recovered from the diseases with uneventful long-term follow-up. These long-term survivors (mean follow-up 7.5 years) have established tolerance, and circulating Tr1 cells are present at high frequency. This study represented the proof-of-concept that Tr1 cells can boost immunotolerance after HSCT, expediting immune recovery while reducing the risk of GvHD. However, higher doses of Tr1 cells may be required to prevent GvHD in a mismatched T-cell replete setting and to obtain a more robust immune reconstitution with infection and relapse prevention. The discovery of DC-10 offered the opportunity to modify the protocol to generate an alloantigen- specific Tr1 cell-rich product. Functional assays demonstrate that stimulation of human PBMCs or CD4^+^ T cells with allogeneic DC-10 induces the differentiation of anergic alloantigen-specific IL-10- producing Tr1 cells (T-allo10) (74). We recently initiated a Phase I clinical trial (NCT03198234) using this improved T-allo10 cell product in the context of mismatched HSCT for children and young adults with hematological malignancies. Purified donor derived CD4^+^ T cells are cultured with tolerogenic DC-10 of host origin in the presence of IL-10 for 10 days to obtain allo-antigen specific Tr1 cells. In this setting, donor-derived T cells should react against host allo-antigens to suppress GvHD after HSCT. Tr1 cells *ex vivo* generated are donor-derived and specific for patient allo-antigens. The current T-allo10 product contains up to 15% of CD49b^+^LAG-3^+^ Tr1 cells, compared to <5% in the first generation IL-10 DLI product. The first results with the lowest dose indicate that the therapy is safe and well-tolerated, while the effects of GvHD and long-term tolerance are yet to be established.

These results suggest that further optimization of Tr1 cell products is required for future clinical studies. The T-allo10 cell product still contains a large proportion of effector T cells that could potentially limit the *in vivo* efficacy of Tr1 cells. To overcome this limitation, a method to generate a selected population of IL-10-producing Tr1 cells by lentiviral vector (LV)-mediated human IL-10 gene transfer was developed. IL-10-engineered CD4^+^ (CD4^IL−10^) cells display a cytokine profile and phenotype super-imposable to *bona fide* Tr1 cells and suppress T-cell responses (76). Adoptive transfer of these human CD4^IL−10^ cells in NSG mice did not result in GvHD, but down regulated GvHD induced by human PBMC. CD4^IL−10^ cells showed GvL effect in mice transplanted with human leukemic cell lines, indicating that these cells have clinical potential for prevention of both GvHD and leukemia relapse after HSCT (77). CD4^IL−10^ cells selectively eliminate CD13^+^ leukemic cells and for optimal killing of target cells, require stable CD54/LFA-1-mediated adhesion and CD112/CD226-mediated activation. This newly identified antileukemic activity of CD4^IL−10^ is a promising area for investigation into identification of treatment regimens that prevent GvHD without affecting the GvL effect.

## Next Steps and Future Perspectives

### αβHaplo-HSCT: The Optimal Platform for Adoptive Immunotherapy?

Despite significant improvement in NRM, leukemia recurrence remains the most important cause of treatment failure in patients with hematological malignancies. This is true for matched-donor HSCT as well as for patients undergoing haplo-HSCT (36). In the latter group, outcomes in children with leukemia not in complete remission at the time of HSCT or beyond second CR have been poor (78). For this reason, a significant challenge in HSCT for hematologic malignancies is the identification of novel strategies for the enhancement of a GvL effect, without increasing incidence of GvHD. Recent development of adoptive immunotherapies for hematologic malignancies, such as the infusion of donor-derived either manipulated or unmanipulated donor lymphocyte infusions, offer some promise for patients after haplo-HSCT (79). Haploidentical transplantation allows immediate donor availability for the collection or generation of additional cells, such as T cells or NK cells, which are capable of enhancing the antitumor effects without being rejected. The absence of pharmacological immunosuppression, typical of T-cell depleted approaches, might facilitate expansion and persistence of cell-based products.

A novel approach to both accelerate the recovery of adaptive immunity and to promote GvL activity simultaneously relies on the use of suicide gene-modified T cells. The administration of donor T cells with a “safety switch” mechanism can help in relapse prevention when administered earlier after HSCT, with the possibility of triggering pharmacologically induced cell apoptosis if severe GvHD occurs. The first method described relating to this approach was based on the insertion of the herpes simplex thymidine kinase suicide gene into T cells (HSV-TK cells) to achieve *in vivo* susceptibility to ganciclovir. A phase I/II multi-center trial (TK007) in adult CD34^+^-selected haplo-HSCT patients showed that post-transplantation infusion of the modified T cells enabled regulation of GvHD, while promoting immune reconstitution (80). The results of a multicenter randomized phase III clinical trial (the TK008 study) to assess the efficacy of HSV-TK^+^ cells in the context of CD34^+^ selected haplo-HSCT for leukemia confirmed improved survival, faster immune reconstitution and efficient prevention of GvHD by suicide gene induction (81). Subsequently, Brenner M. et al. developed an alternative strategy using T cells engineered to express caspase 9 (iC9-T cells), which can be activated via a dimerizing agent. In these engineered T cells, the caspase recruiting domain of the human caspase 9 was modified with a drug binding domain, allowing T-cell elimination after administration of a chemical dimerizing drug, AP1903. The administration of AP1903 dimerizes and activates the caspase 9, which activates downstream caspases, leading to rapid apoptosis (within minutes to hours) (82–84). These iC9-T cells provided rapid immune recovery in 10 pediatric patients (ages 3–17) who received haplo-HSCT. In 5 patients who developed GvHD, iC9-T cells were eliminated within 2 h after AP1903 administration and the GvHD was rapidly resolved without a significant effect on antiviral immune reconstitution (82). Currently, a multicenter US (NCT03301168) and EU (NCT02065869), prospective phase I-II clinical trial using αβhaplo-HSCT followed by addback of donor T cells genetically modified with iC9 safety switch (BPX-501 cells) in patients with malignant or non-malignant disorders is underway. BPX-501 cells are infused on day14 ± 4 after the allograft. No post-transplant pharmacological GvHD prophylaxis is included in this study. Patients who develop GvHD resistant to conventional steroid therapy will receive up to three doses of AP1903 to activate iC9. Preliminary results on a subset of patients with high-risk acute leukemias show a 2-year LFS rate approaching 90% (2018 ASH Annual Meeting, abstract #307, Locatelli F).

**Figure 1:**
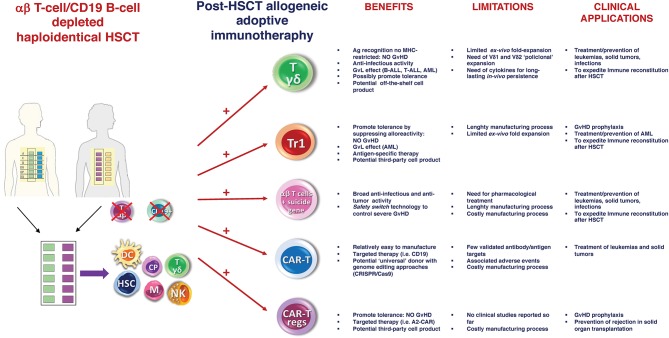
αβ haplo-HSCT as optimal platform for adoptive immunotherapy. Schematic representation of the current and future potential strategies of post-HSCT allogeneic adoptive immunotherapy. Main benefits, limitations and clinical applications of each approach are reported. Ag, Antigen; Tr1, regulatory Type 1 T (Tr1) Cells; MHC, major histocompatibility complex; CAR, chimeric antigen receptor; GvHD, graft-vs-host disease; GvL, graft-vs-leukemia; ALL, acute lymphoblastic leukemia; AML, acute myeloid leukemia; CRISPR, Clustered Regularly Interspaced Short Palindromic Repeats; Cas9, Caspase 9.

Despite these positive preliminary results, the “switch safety” strategy remains a cumbersome approach, requiring time-consuming and costly manufacturing processes. As a result, we are investigating novel strategies based on the infusion of other cell populations post-αβhaplo-HSCT, which may achieve the same goal but are cheaper and easier from a manufacturing standpoint (Figure 1). Specifically, we are focusing on adoptive infusions of *ex vivo* expanded γδ T cells and donor-derived Tr1 cells.

Due to their unique features, γδ T cells are optimally suited for cell therapy in αβhaplo-HSCT when immunogenetic disparity requires both robust anti-leukemia activity and anti-viral protection as well as early immune recovery. As described above, both Vδ1 and Vδ2 γδ T cells can recognize and lyse different type of cancers in a MHC-unrestricted manner, making them good candidates for manufacturing of an off-the shelf cell product. Several clinical trials have highlighted the therapeutic potential of γδ T cells for hematologic malignancies (i.e., Multiple Myeloma and Non-Hodgkin Lymphoma), suggesting that this cell product is both feasible and well-tolerated in addition to its ability to mediate tumor responses (85, 86). Due to the low frequencies of γδ T cells in peripheral blood, an appropriate method of *ex vivo* expansion is required for effective immunotherapy. Some groups are currently investigating the infusion of *ex vivo* expanded γδ T cells to further reduce the risk of disease recurrence without increasing the risk of GvHD (39). Combinations of IL-2 or IL-15 and aminobisphosphonates (i.e., ZOL) are the principal methods currently used for the expansion and activation of these purified γδ T cells.

We have previously described peripheral Tr1 cells, with alloantigen (Allo-Ag)-specific suppressor functions, as being consistently associated with a state of immune tolerance in chimeric patients after allogeneic HSCT. We are currently testing the safety and efficacy of an optimized Tr1-based cell product, the T-allo10 cells, in a phase I clinical trial. In addition to diminish GvHD, T-allo10 cells may also enhance GvL thanks to the cytotoxic activity of activated Tr1 cells (70). An early addback of T-allo10 cells to patients receiving αβhaplo-HSCT could therefore improve immune recovery and decrease relapse.

### Allogeneic T Cells Engineered With a Chimeric Antigen Receptors (CARs) After HSCT: A New Approach?

The engineering of donor lymphocytes to express suicide genes is a security system against the development of severe GvHD. However, it provides a non-targeted antitumor effect. Advances in cell culture and gene transfer technology have resulted in the ability to expand clinically relevant engineered T cells that express chimeric antigen receptors (CARs), which can redirect T cells to recognize a selected target antigen (87). Autologous T cells modified with CD19-targeted CAR constructs consistently demonstrate high antitumor efficacy in children and adults with relapsed CD19^+^ ALL when infused both before and after allogeneic-HSCT (79, 88, 89). Recently, the administration of donor derived CD19-specific CAR T cells early after haplo-HSCT as adjuvant therapy to prevent disease relapse proved to be safe and showed promising results in adult patients with advanced CD19^+^ non-Hodgkin lymphoma or ALL (90). It is reasonable to hypothesize that a similar approach will be applied in the pediatric setting.

The use of autologous cells for manufacturing CAR T cells can be a challenge in patients with malignancies who are often under intensive chemotherapy. One or multiple rounds of apheresis may be required, in addition to the considerable processing time required to isolate, transduce, expand and release the clinical grade products. Patients with active malignancies also often have decreased numbers and reduced functionality of T cells (91). Furthermore, gene transfer leads to a heterogeneous cell population with varying vector copy numbers and expression levels of the CAR. Retroviral or lentiviral engineered T cells also potentially carry the risk of transformation from insertional mutagenesis (92). Therefore, use of CAR T cells both as a bridge before HSCT or as a relapse prevention tool needs to be further investigated.

The advent of genetic engineering using guided endonucleases allows for the introduction of precise modifications in the genome of immune cells by creating specific DNA double-strand breaks, and introducing a homologous DNA template to the targeted region that stimulates DNA repair through homologous recombination. This makes it possible to efficiently disrupt genes and integrate whole transgene cassettes through introduction of a DNA template (93). Use of Clustered Regularly Interspaced Short Palindromic Repeats (CRISPR)-associated guided endonuclease Cas9, complexed with a chemically modified single guide RNA (sgRNA) to form a ribonucleoprotein (RNP), combined with recombinant adeno-associated virus (rAAV) can lead to targeted integration of transgenes in 50–80% of primary human T cells (93).

Gene editing can be used to overcome allo-recognition, which otherwise limits allogeneic T-cell therapies. Initial proof-of-concept applications have included generation of “universal” T cells expressing CARs against CD19 target antigens combined with transient expression of DNA-targeting nucleases to disrupt the T cell receptor alpha constant chain (TRAC) (94). The use of “universal donor” allogeneic T cells to produce CAR T cells might address the challenges associated with the use of patient-derived cells, like decreased T-cell function (91) and contamination with leukemic cells (95, 96). June C. H. et al., recently reported the case of a 20 year-old male with B-ALL treated with tisagenlecleucel (Kimryah®, Novartis) who relapsed after 261 days. Refined immunophenotypic evaluation of the circulating leukemic cells of this patient revealed that they were CAR-transduced B-cell leukemia (95). This finding clearly demonstrated the need for improved manufacturing technologies. Proof-of-principle studies have shown that knock-out of the TCR can prevent T cells from alloreactivity, thereby making it possible to use allogeneic, donor-derived T-cell products without the risk of GvHD (92, 93). Furthermore, targeted integration of the CAR into the constant region of the TRAC in-frame with the endogenous gene's open reading frame has the potential to increase CAR T cell functionality and persistence (97). Ultimately, this technology could enable potent CAR T-cell immunotherapy in combination with allogeneic HSCT, thus abrogating the risk of GvHD.

### FOXP3^+^ Tregs-Based Adoptive Cell Therapy

FOXP3^+^ Tregs are key players in the maintenance of peripheral tolerance in physiological and pathological conditions. FOXP3^+^ Treg-based cell therapies to restore tolerance in T-cell mediated disorders have been extensively investigated (98). Translation to the clinic is difficult, as Tregs represent only 2–10% of CD4^+^ T cells in peripheral blood. The transfer of meaningful numbers requires *in vitro* expansion protocols that are expensive and technically challenging. Nevertheless, GMP grade, large-scale Tregs expansion has been achieved (99), and studies infusing third-party, UCB-derived Tregs as a component of GvHD prophylaxis demonstrate promising results (100). The infusion of Tregs (with or without IL-2) directly isolated from donors, has been tested for the treatment of chronic GvHD. These expansion protocols primarily rely on polyclonal anti-CD3/28 antibody-based expansion. The mechanisms by which Tregs may attenuate GvHD include release of regenerative cytokines (i.e., amphiregulin) (101), APC function inhibition (i.e., via CTLA4), and the inhibition of T effector cells by the release of inhibitory molecules (i.e., adenosine, TGF-β, IL-35, and IL-10) (102) and/or IL-2 consumption (103). Several hurdles, including insufficient number of FOXP3^+^ Tregs and lack of stability and antigen specificity of *in vitro* expanded FOXP3^+^ Tregs, have made clinical use of this cell subset challenging. Recent advances such as LV-mediated gene transfer of the transcription factor FOXP3 in conventional CD4^+^ T cells, to convert effector T cells into Treg-like cells (104), and genetic manipulation to confer antigen specificity and enhance the potency of FOXP3^+^ Tregs (105), offer new avenues to make adaptive Treg cell therapy more feasible and effective.

We developed a LV-based strategy to ectopically express high levels of FOXP3 that do not fluctuate with the state of T-cell activation. This method produces suppressive cells that are as potent as *ex vivo* isolated FOXP3^+^ Tregs and can be propagated as a homogeneous population. Using this system, both naïve and memory CD4^+^ T cells can be efficiently converted into Tregs (106). We further explored the adoptive transfer of *in vitro* engineered autologous Tregs to control autoimmunity in patients with immune-dysregulation, polyendocrinopathy, enteropathy, X-linked (IPEX) syndrome caused by mutations in FOXP3 (107). The human FOXP3 coding sequence was cloned under the control of a constitutive promoter in a bidirectional LV construct allowing simultaneous expression of full-length FOXP3 and of a cell-surface marker (ΔNGFR) for the identification/selection of transduced T cells (LV-FOXP3). CD4^+^ T cells converted into FOXP3^+^ Treg cells by LV-mediated FOXP3 gene transfer (CD4^FOXP3^) display a stable phenotype and suppressive function and are stable in inflammatory conditions *in vitro* and in a model of xenogeneic GvHD (107). These findings pave the way for the treatment of IPEX patients by adoptive cell therapy using genetically engineered Treg cells. These data also lay the foundation for future use of CD4^FOXP3^ T cells to prevent or treat GvHD after HSCT. The fact that CD4^FOXP3^ T cells can be obtained from naïve or memory CD4^+^ T cells renders the manufacturing process easier and more cost effective compared to other methods. CD4^FOXP3^ T cells do not require extensive *in vitro* expansion with high cytokine concentration. Once safety and proof-of-concept studies are completed in IPEX patients, use of these Treg-like cells could be investigated as a treatment for severe GvHD in the context of allogeneic HSCT.

The successful use of effector T cells carrying specific CARs suggests that a similar approach can be applied to generate alloantigen-specific Tregs. Levings M. K. et al., recently reported the creation of HLA-A2-specific CAR (A2-CAR) Tregs (105). *In vitro*, A2-CAR-expressing Tregs maintain their expected phenotype and suppressive function before and after A2-CAR mediated stimulation. In a xenogeneic GvHD animal model, human A2-CAR-expressing Tregs were superior at preventing xeno-GvHD caused by HLA-A2^+^ T cells to Tregs expressing an irrelevant CAR. These results suggest that the CAR technology can be used to generate alloantigen-specific human Tregs, enhancing their therapeutic potential in HSCT.

Recently, the concept of third-party Treg cell therapy has also emerged with the aim of improving safety, quality, accessibility and cost. As for effector T cells, wide application of adoptive Treg cell therapy using autologous T cells might be limited due to its nature as a patient-specific cell product, which is time-consuming and expensive to manufacture. The use of third-party Tregs also offers the opportunity to isolate cells from sources other than peripheral blood. Bone marrow or UCB could be used instead. However, third-party cells express allogeneic HLA molecules, which render them susceptible to rejection by the recipient's immune system. To overcome this hurdle, several research groups have proposed using genome editing technology to knockout β-2-microglobulin or FASL to generate HLA class I negative T cells or FAS-resistant T-cells, respectively (108). Furthermore, the use of the CRISPR/Cas9 system to knockout the endogenous TCR and generate TCR negative cell products could represent a solution to the rejection issue.

## Conclusions

Allogeneic HSCT has dramatically changed the natural course of hematological malignant and non-malignant diseases and provided a definitive cure for many patients. Transplanted patients have offered a unique opportunity for investigating tolerance establishment across the donor/recipient allogeneic barrier. Limited availability of fully matched related and unrelated donors has promoted the search for new strategies of graft engineering to overcome the HLA mismatch of haploidentical donors. In this setting, αβ T-cell/CD19 B-cell depletion has shown the most robust and promising clinical results in pediatric patients. Due to the absence of post-HSCT pharmacological prophylaxis, αβhaplo-HSCT might represent the optimal platform for adoptive immunotherapy. In addition to the established strategy using “safety switch” cells, new approaches using post-HSCT infusion of *ex vivo* expanded T-cell subpopulations (i.e., γδ T cells and Tr1 cells) or engineered autologous or allogeneic T cells (i.e., CAR T cells) represent the future of immunotherapy for the optimization of hematological malignancies treatment. To facilitate and accelerate the clinical implementation of T effector- and Treg-based cell therapy, effective and cost-efficient GMP-grade protocols must be established in order to generate the large numbers of cells required for successful patient treatment. Methods of efficient homing and entry of effector T cells into tissues (109) will also be critical to the clinical success of these adoptive immunotherapy approaches. This will require further understanding of the mechanistic cellular and molecular components impacting trafficking of T effector cells (109).

## Author Contributions

AB and MR equally contributed in conceiving, writing, and editing this manuscript.

### Conflict of Interest Statement

The authors declare that the research was conducted in the absence of any commercial or financial relationships that could be construed as a potential conflict of interest.
